# Progress in Functional Urology Reflected in Recent Papers in the *Journal of Clinical Medicine*

**DOI:** 10.3390/jcm12134482

**Published:** 2023-07-04

**Authors:** Martin C. Michel

**Affiliations:** Department of Pharmacology, University Medical Center, Johannes Gutenberg University, 55131 Mainz, Germany; marmiche@uni-mainz.de

**Keywords:** benign prostatic hyperplasia, overactive bladder syndrome, stress urinary incontinence, epidemiology, pathophysiology, diagnostics, pharmacological treatment, physical agent-based therapy, surgery

## Abstract

Benign conditions of the lower urinary tract, including benign prostatic hyperplasia, overactive bladder syndrome, and stress urinary incontinence, are frequent in the general population. Despite their benign nature, they have major adverse effects on the quality of life of the afflicted patients and their partners. Despite major progress in the diagnosis and treatment of these conditions, improved understanding and management of these patients remain substantial medical needs. This editorial discusses some recent high-quality articles published in the *Journal of Clinical Medicine* on the understanding of the epidemiology, pathophysiology, diagnostic, and treatment of benign diseases of the lower urinary tract tissues such as the bladder and prostate.

## 1. Introduction

Conditions such as overactive bladder syndrome (OAB), stress urinary incontinence (SUI), or male lower urinary tract symptoms (LUTS) suggestive of benign prostatic hyperplasia (BPH) are frequent in the general population and adversely affect the quality of life (QoL) of the afflicted patients and their partners [1]. Interstitial cystitis/bladder pain syndrome (IC/BPS) occurs less frequently than the other three mentioned conditions but tends to have a greater individual impact. While major advances have been made in the understanding and treatment of conditions such as OAB and related functional disorders, medical needs continue to exist because available treatments are insufficiently effective or tolerated in some patients, leading to poor long-term persistence on a treatment regimen [2]. Against this background, this article discusses some recent studies in the field of functional urology and how they contribute to our understanding of these conditions, with a focus on those reported in the *Journal of Clinical Medicine*. While the selection of articles discussed here is subjective, they will hopefully be of interest to many working in this field. This discussion is organized based on the studied topic: pathophysiology, diagnostics, and treatment.

## 2. Epidemiological Studies in Functional Urology

The general epidemiology of OAB and BPH has been the subject of many studies. A nationwide cohort study from Sweden including more than two million women aged 15–50 years was used to obtain insight into the sociodemographic of urinary incontinence [3]. A Cox regression analysis was performed to explore the association of various sociodemographic variables with time to a first diagnosis of incontinence. The incidence rate of incontinence in this relatively young population was 1.85 per 1000 person-years. In the fully adjusted model, a greater age, lower education, and being born outside of Sweden were independently associated with a greater risk for incontinence. The incidence rates of women born in the Middle East/North Africa and in Latin America/Caribbean were 2.41 and 2.30, respectively. Parity was strongly and independently associated with the incidence of incontinence, most likely reflecting the known role of vaginal delivery in the pathophysiology of SUI. Interestingly, income had only a minor impact on incontinence, and living in a rural area was associated with a lower incidence. Some of these risk factors had previously been unknown.

A population-based study from Taiwan performed a nested case–control study on the National Longitudinal Health and Insurance Database to explore the associations between stressful medical conditions and the occurrence of IC/BPS [4]. Applying multiple conditional regression, 1103 IC/BPS patients and 4412 non-IC/BPS patients were compared. Stress-related diseases preceded the diagnosis of IC/BPS by 3 months to 2 years. Increases in the statistically significant risk for IC/BPS were observed for urinary tract infection (OR 10.95) and, with weaker associations, chronic obstructive pulmonary disease (OR 1.48), peptic ulcer (OR 1.69), inflammatory bowel syndrome (OR 1.66), autoimmune disease (OR 1.48), depression (OR 1.54), sleep disorders (OR 1.45), and allergic rhinitis (OR 1.29). As a diagnosis of IC/BPS is largely made by exclusion and often occurs only several years after the onset of symptoms, knowledge of such associations may help in obtaining an earlier diagnosis and treatment, leading to less suffering.

## 3. Pathophysiology Studies in Functional Urology

Dysfunction of the pelvic floor plays a role in the pathophysiology of SUI, and vaginal child delivery is one of the most important risk factors for developing SUI. Some investigators from Spain performed a cross-sectional study in 64 primiparous women at 8 weeks after delivery and applied a transabdominal ultrasound during movements that activate the abdominopelvic cavity musculature [5]. While voluntary contraction of the pelvic floor musculature caused the bladder base to ascend in all but one participant, activation of the transverse abdominis muscle caused the bladder base to ascend in 56% of women and to descend in the others. Coughing, causing an elevation in intraabdominal pressure, caused a descent in all participants. The authors proposed that function of the transverse abdominis muscle should routinely be assessed to enable early intervention in postpartum women at risk of developing SUI.

Increases in intraabdominal pressure can occur during coughing or in patients with obstructive lung disease during forced breathing, which may provoke episodes of SUI. A corresponding avoidance behavior could weaken expiratory muscles. A team of investigators from Tunisia, in collaboration with colleagues from Australia and several European countries, investigated the associations between respiratory muscle strength, physical function, and SUI in 31 women with SUI in comparison with 29 women without SUI [6]. Women with SUI had more body fat, a greater body mass index and waist circumference, and weaker postural gait and abdominal muscles. Respiratory muscle strength exhibited a moderate inverse correlation with SUI severity. The authors proposed applying pelvic floor muscle training to increase confidence in increasing the intraabdominal pressure and thereby strengthening expiratory muscles and eventually improving overall physical function.

While the idea that urine from healthy people is sterile has been abandoned several years ago, the idea that the colonization of the lower urinary tract may play a pathophysiological role in disease is relatively new. Some investigators from Poland reviewed the existing evidence for a role of dysbiosis of the urinary microbiome in the pathophysiology of OAB [7]. They reported on a growing body of evidence that qualitative and quantitative characteristics of the individual urinary microbiome are related to OAB symptoms in adults. The evidence in adolescents appears more limited, possibly reflecting that the occurrence of OAB is age-related. Given that all presently available treatments of OAB are symptomatic only [2], a better understanding of the role of the urinary microbiome, at least theoretically, may lead to curative treatments.

Some Austrian and Swiss investigators provided a case–control study mapping the urinary proteomic profile of 20 women each with and without OAB [8]. They identified 1897 proteins, of which 37 in the OAB and 53 in the control group merited further analysis. They were mainly involved in pathways related to cellular responses to stress and apoptosis in the OAB group, whereas those in the control group were mainly related to immunological, microbial-protective, and tissue-elasticity processes. As OAB is a symptomatic diagnosis, it has been argued that the lack of major progress in this field may largely be due to the heterogeneity of patients receiving this diagnosis [9]. Studies like that from Koch et al. may help to objectively identify subsets of OAB patients with a defined pathophysiology that may give rise to more targeted treatments for such subgroups.

It has been established several years ago that the urine of OAB patients contains higher concentrations of neurotrophins such as nerve growth factor compared with that of the controls. However, while of pathophysiological interest, proposals that urinary neurotrophin concentrations may be biomarkers of OAB have been met with skepticism [10]. Some investigators from Poland have now investigated the effects of transcutaneous electrical nerve stimulation in 30 children aged 5–12 years with OAB in comparison with 30 children without OAB [11]. While treatment was associated with a decrease in urinary neurotrophins, which depended on age and presenting symptoms, the levels before and after treatment were considerably higher than those in the controls without OAB. Exhibiting only limited responsiveness to treatment adds to the doubts that the urinary levels of neurotrophins are meaningful biomarkers of OAB.

## 4. Studies of Diagnostics and Patient Management in Functional Urology

LUTS can occur idiopathically but are secondary to neurologic damage in many patients as it can occur in the context of diseases of the central nervous system such as multiple sclerosis (MS) or spinal cord injury and those of the peripheral nervous system such as diabetic neuropathy. While many studies have addressed the epidemiology of OAB in general, more limited information is available for neurological dysfunction of the lower urinary tract, e.g., in MS, although about two thirds of MS patients suffer from LUTS and sexual dysfunction. An interdisciplinary review from Italian neurologists, urologists, and gynecologists described the large variability of symptoms in MS patients [12]. There appears to be a general correlation between the severity of the central nervous manifestations of MS and of LUTS. The interdisciplinary panel of authors recommends specific roles for urologists, neurologists, and gynecologists in the management of MS patients and their LUTS. As many patients with LUTS do not actively address their urogenital complaints out of shame, physicians managing MS patients should actively assess LUTS to enable early intervention for an adequate management.

Relative to the general population, OAB occurs more often in some conditions, including liver cirrhosis. One plausible underlying mechanism in this group of patients is fluid redistribution resulting from portal hypertension and ascites. Against this background, some investigators from Taiwan analyzed Overactive Bladder Symptom Score data from 158 patients with chronic viral hepatitis-related liver cirrhosis [13]. OAB in general and OAB-wet in particular were present in 31% and 18%, respectively, of cirrhosis patients in this cohort. Patients with a score of ≥2 had about 20% lower platelet counts regardless of the use of diuretics. Subjects with nocturia or with urgency incontinence had slightly lower serum albumin levels than those without. Gender; presence of ascites, diabetes, or hypertension; or use of diuretics were not associated with OAB in general, nocturia, or urgency incontinence. Whether platelet counts and serum albumin levels are associated with OAB in patients without liver cirrhosis remains to be determined.

## 5. Studies on Treatment in Functional Urology

Several medical OAB treatments are available in multiple dose strengths. Only limited information is available about factors associated with the initial choice of starting dose and dose escalation. Moreover, most of the limited evidence comes from randomized controlled trials that, due to their inclusion and exclusion criteria and the artificial setting of being part of a clinical study, may have limited external validity. Therefore, a group from Germany analyzed two large non-interventional studies (1335 and 745 patients) of similar design in which patients receiving 30 and 45 mg/d propiverine as OAB treatment were followed for at least 12 weeks [14]. While several factors were associated with initial dosage choice, dose escalation, or treatment outcomes after 12 weeks based on a multivariate analysis within each of the two datasets, only a few were consistent across both studies. This highlights that even large datasets do not always yield robust findings. The only consistent factor associated with a higher starting dose was younger age. Consistent factors associated with a dose increase after 4 weeks included basal number of urgency episodes and changes in incontinence episodes. Treatment outcomes as assessed by symptom intensity differences between baseline and study end for each OAB symptom were strongly driven by baseline values. Interestingly, patients starting on 30 mg and escalating to 45 mg after 4 weeks had comparable outcomes to those starting at and staying on 30 or 45 mg, indicating that dose escalation is a meaningful approach in the management of OAB patients.

Non-pharmacological options are routinely applied in patients with LUTS in whom pharmacological approaches are insufficiently effective and/or tolerated. A Swiss–Italian collaboration has applied controlled nerve depolarization resulting in sacral root S2–S4 neurostimulation and pelvic muscle contraction in 40 and 60 women with OAB or SUI, respectively [15]. This was applied via functional magnetic stimulation with patients sitting in a special chair that generates a 3 Tesla field (two 20 min sessions per week for 8 weeks). The primary outcome parameters of this non-controlled study were the PGI-I and a patient satisfaction scale. After two months, 47% of SUI and 50% of OAB patients subjectively reported being cured. An additional 21% and 20% reported improvement. While these data appear promising, additional data on longer term outcomes and sham-controlled data are necessary for a full evaluation of this technique.

However, electromagnetic stimulation is not the only non-pharmacological option for the treatment of OAB. Already well-established options include sacral or tibial nerve neurostimulation, which are currently considered third-line treatment options. Some Taiwanese investigators have reviewed such options and concluded that these have high efficacy with minimal side effects [16]. The limitations of neuromodulation appear to be the need for implantation; although they can be cost-effective in the long term, the upfront cost for the device and its implementation can be high.

While medical treatment is often the preferred initial approach to the management of BPH, some patients, primarily after failure of medical treatment, undergo surgical approaches such as transurethral resection of the prostate (TURP). While TURP is considered to be the gold standard of relieving obstruction, it can have complications. A population-based, retrospective cohort study from Taiwan has compared the incidence of emergency room visits and rehospitalization for bleeding complications following TURP or laser-based transurethral procedures for the treatment of BPH [17]. Using data from the Taiwan National Health Insurance Research Data Base, propensity score matching, and applying a multiple regression analysis, they looked at 1651 patients each receiving laser or TURP treatment between January 2015 and September 2018. Diode laser enucleation had a hazard ratio of 1.5 relative to TURP for an emergency room visit due to clot retention as compared with monopolar TURP. However, GreenLight laser photovaporization and thulium laser vaporesection had hazard ratios of 0.61 and 0.67, respectively, as compared with TURP. These data indicate that various laser-based techniques differ in their risk of clot retention, leading to emergency room visits. Such data should be considered in the choice of benefit/risk ratios during the selection of the optimal surgical approach to BPH.

Bladder neck contraction is a possible complication following endoscopic treatment of BPH. A German group conducted a chart review of 60 men undergoing transurethral treatment for bladder neck contraction following prior endoscopic BPH treatment [18]. Ninety percent of patients had previously undergone TURP and ten percent had undergone holmium laser resection. They reported that a longer time interval between previous BPH treatment and subsequent bladder neck contraction, and prior use of a holmium laser seem favorable for successful contraction treatment. However, this conclusion requires confirmation in larger cohorts.

In benign conditions such as BPH, OAB, and SUI, patient satisfaction with treatment outcomes matters most. Some Japanese investigators performed a cross-sectional, web-based survey of 1004 OAB patients aged ≥50 years who had received OAB medication in the past 2 years [19]. At the time of the survey, 58% were on a treatment and 24% had discontinued treatment for reasons other than symptom improvement. According to a multivariate analysis, treatment effectiveness was associated with patient–physician communication, female gender, and not switching between medications. It remains unclear whether the latter reflects that not switching leads to more effectiveness or greater perceived effectiveness leads to not switching medications. The latter appears more likely as reported improvements in symptoms and patient satisfaction were greater among subjects remaining on their treatments.

## 6. Conclusions

The current selection of recently published articles demonstrates that the *Journal of Clinical Medicine* is an attractive home for high-quality studies on the epidemiology, pathophysiology, diagnostics, and treatment in functional urology. It exposes high-quality findings in this area to an interdisciplinary global readership. The authors publishing such studies in this journal are also a geographically highly diverse group.
